# Vertical Strength Transfer Phenomenon Between Upper Body and Lower Body Exercise: Systematic Scoping Review

**DOI:** 10.1007/s40279-024-02039-8

**Published:** 2024-05-14

**Authors:** Ivan Curovic, David Rhodes, Jill Alexander, Damian J. Harper

**Affiliations:** 1 Institute of Coaching and Performance, School of Health, Social Work and Sport, University of Central Lancashire, Preston, UK; 2 Human Performance Department, Burnley Football Club, Burnley, UK; 3 Jurija Gagarina 102/7, 11070 Belgrade, Serbia

## Abstract

**Background:**

There are a myriad of exercise variations in which upper body (UB) and lower body (LB) exercises have been intermittently used. However, it is still unclear how training of one body region (e.g. LB) affects adaptations in distant body areas (e.g. UB), and how different UB and LB exercise configurations could help facilitate physiological adaptations of either region; both referred to in this review as vertical strength transfer.

**Objective:**

We aimed to investigate the existence of the vertical strength transfer phenomenon as a response to various UB and LB exercise configurations and to identify potential mechanisms underpinning its occurrence.

**Methods:**

A systematic search using the PRISMA (Preferred Reporting Items for Systematic Reviews and Meta-Analyses) for Scoping Reviews protocol was conducted in February 2024 using four databases (Web of Science, MEDLINE, Scopus and CINAHL) to identify peer-reviewed articles that investigated the vertical strength transfer phenomenon.

**Results:**

Of the 5242 identified articles, 24 studies met the inclusion criteria. Findings suggest that the addition of UB strength training to LB endurance exercise may help preserve power-generating capacity for the leg muscle fibres. Furthermore, systemic endocrine responses to high-volume resistance exercise may beneficially modulate adaptations in precedingly or subsequently trained muscles from a different body region, augmenting their strength gains. Last, strength training for LB could result in improved strength of untrained UB, likely due to the increased central neural drive.

**Conclusions:**

Vertical strength transfer existence is enabled by neurophysiological mechanisms. Future research should involve athletic populations, examining the potential of vertical strength transfer to facilitate athletic performance and preserve strength in injured extremities.

## Key Points

**Table:** 

Upper body strength training positioned alongside a running or cycling session may yield beneficial systemic effects for the leg muscle fibres.
High-volume resistance exercise of an entire body segment (e.g. lower body) may facilitate strength training adaptations in a subsequently trained single muscle group from the other body segment (e.g. elbow flexors).
High-volume resistance exercise of the lower body musculature may facilitate strength training adaptations in previously or concurrently trained multiple muscle groups from the upper body.
Lower body strength training alone may increase strength in the untrained upper extremities, while the opposite transfer direction (i.e. upper body to lower body) has not been sufficiently explored.

## Introduction

Various resistance training strategies are used to support athletic performance [1, 2] and accelerate return to sport after injury [3]. These strategies take advantage of different exercise modes utilising mixed loads and high-velocity contractions with the goal to increase power production [2, 4], prevent injury occurrence [5] and preserve muscle mass in players recovering from injury [6]. To ensure optimal health and performance of professional athletes, it is crucial to implement effective resistance exercise conditioning [7–10]. Importantly, the changes that occur in upper body (UB) and lower body (LB) muscles differ in response to the similar exercise modes [11–14]. Furthermore, it appears that concurrent training of both body segments elicits favourable endocrine responses compared with the training of a single body region [15–17], which could lead to improved functional outcomes in both regions [16, 18], regardless of the combination type.

Unlike cross-education, where the confirmed transfer of strength dissipates from the trained to the untrained limb in the same body compartment (e.g. LB) [19, 20], a cross-transfer between UB and LB exercise only recently appeared as a subject of more thorough investigations [21–23]. The significance of this phenomenon is reflected in: (a) recorded strength improvements of untrained muscles from one body region (e.g. upper extremities) resulting from exercise of another body region (e.g. leg muscles) [22], and (b) enhanced strength adaptations of strength-trained muscles from the targeted region (e.g. elbow flexors) facilitated by concurrent high-volume exercise of remote muscle groups (e.g. leg muscles) [24]. While former training adaptation could be labeled as “vertical strength transfer” (VST), latter adaptations are likely enabled via exercise-induced endocrine responses [24], and may be more accurately described as “vertical strength facilitation”. Nevertheless, for the purpose of this review, both cross-effects will be referred to as VST, regardless of whether strength enhancement was caused or facilitated by exercise of a remote body segment.

Circulating endocrine factors obtained by various training modes can systemically modulate different tissues and neurons [25–29], thereby influencing exercise adaptations on a whole-body level. For example, an acute bout of metabolic pre-conditioning with “all out” running has been shown to successfully increase oxidative metabolism and performance in subsequent anaerobic UB strength-endurance exercise [30], potentially leading to enhanced chronic adaptations. These humoral factors secreted into circulation by the exercised muscles (i.e. “exerkines”) [29, 31] are not only limited to the positive influence on overall health [32], but have been suggested to contribute to strength improvements of the untrained muscle groups in older adults [31]. Unfortunately, exact mechanisms are yet to be elucidated. Moreover, neurological effect may be realised via increased “voluntary activation” with strengthened neural impulses from the motor cortex to the motoneuron pool and from the motoneuron pool to the muscle fibres [33]. For instance, Da Silva et al. [34] recorded successful post-activation performance enhancement for the horizontal jumping task in female soccer players after UB pre-conditioning activity with heavy bench pressing, suggesting the role of the central nervous system (CNS) for high-intensity contractions in unrelated body areas. This effect may be transferred to the long-term strength improvements on the account of an intensified central neural drive [33].

The understanding of the crosstalk between separate muscle tissues and motor neurons may aid in more successful manipulations of training strategies and result in an augmentation or preservation of the strength or power gains in the targeted muscles. One such example is concurrent UB resistance exercise and LB endurance exercise mode for which research has mostly focused on the extent of the negative impact towards UB strength development [35–37]. However, this combined training regime may yield advantageous morphological adaptations for the leg muscle fibres [38], preserving their size from decreasing after long running sessions [39], potentially leading to improved power expression in LB [38]. Furthermore, resistance training of LB muscles may lead to strength improvements in upper extremities [18], regardless of whether they were active or not [22], which could be critical during the injury recovery periods. Therefore, the purpose of this scoping review was to investigate the existence of the VST phenomenon as a response to various UB and LB exercise configurations. To help identify potential mechanisms that may underpin VST, we included studies that investigated acute exercise responses with immediate effect on remote muscle tissues.

## Methods

### Experimental Approach to the Problem

Scoping reviews are carried out to identify and analyse the latest concepts within a specific research field [40]. These reviews differ from standard systematic reviews because they address broader and less defined research questions for which the availability of relevant studies may be less clear [41]. Therefore, to allow the examination of a broad range of literature with the goal of evaluating the existence of the VST in various training protocols, the Preferred Reporting Items for Systematic Reviews and Meta-Analyses (PRISMA) for Scoping Reviews extension was followed [42]. A systematic search of four electronic databases (Web of Science, MEDLINE, Scopus and CINAHL) was conducted in February 2024 by the lead author using the default fields search setting within each database. Only original full-text peer-reviewed articles written in English were considered with no restrictions on year of publication. Search terms were as follows: “cross transfer training” OR “strength training” OR “unilateral training” AND “cross-transfer effect” OR “physiological effects” OR “systemic effects” OR “neuromuscular adaptation”.

### Study Selection Criteria

After eliminating duplicates and removing records following abstract screening, search results were independently screened by two reviewers using the inclusion and exclusion criteria. Any discrepancies were resolved through discussion (*n* = 7). Studies eligible for inclusion were those that investigated: (1) concurrent LB endurance and UB resistance exercise (acute or intervention) effect on LB muscle adaptive potential (morphology, strength and power); (2) LB resistance exercise (acute or intervention) effect on concurrently trained UB muscle adaptive potential (morphology, strength and power) or vice versa; and (3) LB resistance exercise intervention effect on untrained UB strength or vice versa.

Studies were excluded from the review if they: (1) paired LB endurance (i.e. running or cycling) with UB resistance exercise without examining LB adaptive potential; (2) had insufficient duration (< 5 min) of the LB endurance protocol (i.e. running or cycling) to be considered an independent exercise session (e.g. pre-workout warmup); (3) focused on a unilateral training adaptation limited to the cross-education phenomenon only (i.e. training of one limb influencing contralateral limb); (4) applied an exercise intervention that did not distinguish between the two body segments (i.e. weightlifting); and (5) applied an exercise intervention that was not standardised between the training groups.

### Data Extraction

All data for each study were extracted by the lead author (IC), including: (1) general characteristics (year of publication, sample population, sample size); (2) participant characteristics (sex, age, body mass, height); (3) aims of the study; (4) details of the intervention; (5) outcome measures, and (6) key findings (Tables 1, 2, 3). Following initial data extraction, a random subset of studies was assigned to one other author (DH) to check the accuracy of extracted data. This approach ensured the reliability of the collected information, minimising errors or discrepancies in the dataset.

**Table 1:** Vertical strength transfer effects between upper body resistance exercise and running or cycling session

Study	Participant characteristics					Training protocol			Key findings	Vertical strength transfer
	Population	*n*	Age (years)	Stature (cm)	Body mass (kg)	Duration	Intervention	Outcome measures of interest		
Kraemer et al. [38] (1995)	Healthy male individuals	35	22.5 ± 4.2	SG: 175.3 ± 6.1 EG: 177.6 ± 7.8 SEG: 174.1 ± 6.4 USEG: 176.7 ± 4.0 CG: 176.5 ± 7.0	SG: 76.6 ± 14 EG: 75.3 ± 6.7 SEG: 74.2 ± 6.7 USEG: 75.6 ± 8.5 CG: 76.2 ± 5.4	12 weeks, 4 × per week	SG vs EG vs SEG vs USEG vs CG	1-RM bench press (kg), 1-RM military press (kg), Wingate anaerobic test (W), vastus lateralis muscle fibre morphology (μm^2^), leg muscle fibre area Testosterone (nM), and cortisol (nM) measured at the 4th, 8th, and 12th week of training	UB resistance training prevented leg power loss evident in EG (peak power -8 W, mean power -14 W on average) and resulted in small power increases (peak power + 39 W, mean power + 20 W on average) UB resistance training prevented significant decrease in type I and type IIc fibre area of vastus lateralis muscle that were evident in EG EG showed increased cortisol levels and no changes in testosterone, while USEG showed no changes in testosterone-to-cortisol ratio	Vertical strength transfer from UB strength training to LB muscle fibres preserving the power-generating capacity after long running sessions
Verney et al. [55] (2006)	Active older male individuals	10	73 ± 4 years	164–172	71.3 –84.1	14 weeks, 3 × per week	One group: UB strength training interspersed by 3 × 12 min of HIIT cycling for endurance	Leg isometric and isokinetic torque (Nm), UB and LB muscles’ CSA (cm^2^)	Maximal isometric and isokinetic torque for knee extension increased significantly by 13% (*p* = 0.01), and 9% (*p* = 0.03), 12% (*p* = 0.005) and 10% (*p* = 0.01) at 30°, 60° and 120° s − 1, respectively A significant increase in CSA for UB muscles (range 5–14%), but not in hamstring muscles. Muscle CSA increased by 2% in the quadriceps muscle	Vertical strength transfer from UB strength training to LB strength improvement after concurrent cycling sessions
Verney et al. [16] (2008)	Active older male individuals	10	73 ± 4 years	164–172	71.3–84.1	14 weeks, 3 × per week	One group: UB strength training interspersed by 3 × 12 min of HIIT cycling for endurance	Leg fibre morphology (μm^2^), leg fibre percentage, number of NCAM + satellite cells per fiber	A significant 38% increase in the number of NCAM + satellite cells per fiber in both deltoid (*p* = 0.03) and vastus lateralis (*p* = 0.05) muscles A significant increase in the number of NCAM + cells associated with type II fibers (+ 58%, *p* = 0.04, and + 73%, *p* = 0.03, in deltoid and vastus lateralis, respectively) A significant increase of fibre area only in type IIa fibres of vastus lateralis (+ 13%, *p* = 0.03)	Vertical strength transfer with modified satellite cells per fibre for both endurance-trained vastus and resistance-trained deltoid muscle
Moberg et al. [17] (2021)	Trained male individuals	8	31 ± 5	182 ± 5	80 ± 5	Acute study protocol	One group with two different protocols on separate days: arm-resistance training vs arm-resistance training preceded by HIIT leg cycling (5 × 4 min high intensity and 25-min low intensity)	Various molecular responses (PGC-1α1, PGC-1α4, MuRF-1 mRNA) extracted from plasma and arm muscle biopsies	Systemic molecular changes by HIIT leg cycling and arm exercise with positive remodelling influence for both regions	Vertical strength transfer potential via beneficial systemic responses by concurrent LB cycling and UB resistance exercise

*CG*  control group, *CSA* cross*-*sectional area, *EG* cardio-respiratory endurance training group, *HIIT* high-intensity interval training, *LB* lower body, *min* minute, *LSG* leg strength training group, *RM* repetition maximum, *SEG* whole-body strength and cardio-respiratory endurance training group, *SG* whole-body strength training group, *UB* upper body, *USEG* UB strength and cardio-respiratory endurance training group

**Table 2:** Vertical strength transfer effects between upper body resistance exercise and lower body resistance exercise

Study	Participant characteristics					Training protocol			Key findings	Vertical strength transfer
	Population	*n*	Age (years)	Stature (cm)	Body mass (kg)	Duration	Intervention	Outcome measures of interest		
Kraemer et al. [45] (2004)	Untrained physically active female individuals	85	23.1 ± 3.5	SG (3–8 RM: 163.7 ± 7.5; 8–12 RM: 165.4 ± 5.4) UB- only training groups (3–8 RM: 165.5 ± 7.2; 8–12 RM: 166.7 ± 6.2) Control group: 164.5 ± 6.2	SG (3–8 RM: 64.1 ± 8.8; 8–12 RM: 63.2 ± 7.0) UB- only training groups (3–8 RM: 66.7 ± 10.7; 8–12 RM: 65.7 ± 12.1) Control group: 65.9 ± 11.2	24 weeks, 3 × /week	SG vs UB-only training groups vs CG	1-RM bench press (kg), 30% of 1-RM ballistic bench press (W), mid-upper arm CSA (cm^2^)	UB power improvement in 3–8 RM SG significantly greater than in other groups (delta change + 68 W, *p* < 0.05) Increases in all three arm muscles (brachialis, biceps brachii, and triceps brachii) only in the 3–8 RM SG	Vertical strength transfer noted by the combination of UB and LB high-resistance exercise sessions leading to increased power production in UB muscles and more significant upper limb hypertrophy
Walker et al. [53] (2004)	Healthy male individuals	20	31.3 ± 4.9	182.7 ± 5.9	From 91.2 ± 13.6 before to 92.1 ± 13.9 kg, *p* < 0.05 after the programme	10 weeks, 2 × /week	SG vs arms-only training group	1-RM elbow flexion (kg), 80% of pre-training 1-RM elbow flexion (free RM), upper arms CSA (cm^2^) IGF-1 (ngmL¯^1^), myostatin (kDa)	No significant differences in resting plasma IGF-1, myostatin, arm strength and arm size between the groups. Myostatin levels (negative modulator of muscle mass) similarly decreased with the UB-only training (20%)	No vertical strength transfer facilitated for the targeted UB muscles trained alongside leg resistance sessions that failed to produce additional systemic factors
Hansen et al. [24] (2001)	Untrained male individuals	16	24.4 ± 3.1	Unilateral arm training group: 181 ± 4.0 Unilateral arm training group followed by leg training: 179 ± 8.0	Unilateral arm training group: 78.2 ± 8.0 Unilateral arm training group followed by leg training: 81.1 ± 25.2	9 weeks, 2 × /week	Unilateral arm strength training group vs unilateral arm strength training followed by leg resistance exercise group	Elbow flexors MVIC (Nm) Testosterone (nM), cortisol (nM), and GH (mg/l) during the first and last session	Significant increases in isometric strength of both the trained arm (37%) and control arm (10%) for the unilateral arm followed by leg training group Cortisol, testosterone, and GH higher in leg-training group	Vertical strength transfer facilitated for the targeted UB muscles trained before leg resistance sessions that produced systemic factors Vertical strength transfer noted for the untrained upper limb after leg resistance sessions
Madarame et al. [45] (2008)	Untrained male individuals	15	21.6 ± 2.4	Unilateral arm training followed by BFR-leg training group: 171.0 ± 3.7 Unilateral arm training followed by normal leg training group: 168.7 ± 4.2	Unilateral arm training followed by BFR-leg training group: 58.8 ± 3.8 Unilateral arm training followed by normal leg training group: 60.7 ± 5.1	10 weeks, 2 × /week	Unilateral arm strength training followed by BFR-leg resistance exercise group vs unilateral arm strength training followed by normal leg resistance exercise group	Maximal isometric elbow flexors torque (Nm), elbow flexors CSA (cm^2^) Noradrenaline (nM), testosterone (nM), GH (ng/ml)	Significant hypertrophy and isometric strength improvement in the trained arm (*p* < 0.05) only for the BFR-leg training group No changes in the untrained arm Noradrenaline concentration–time curve significantly higher in the BFR-leg training group (182.2 ± 79.0 nM for 30 min) compared to the normal exercise group (79.4 ± 16.0 nM for 30 min) (*p* < 0.05)	Vertical strength transfer facilitated for the targeted UB muscles trained before leg resistance sessions that produced systemic factors
May et al. [47] (2018)	Recreationally active male individuals	24	22.6 ± 3.3	Unilateral arm training followed by BFR-leg training group: 177.5 ± 9.5 Unilateral arm training followed by normal leg training group: 174.1 ± 6.7	Unilateral arm training followed by BFR-leg training group: 73.0 ± 13.6 Unilateral arm training followed by normal leg training group: 72.4 ± 11.2	7 weeks, 3 × /week	Unilateral arm strength training followed by BFR-leg resistance exercise group vs unilateral arm strength training followed by normal leg resistance exercise group	1-RM elbow flexion (kg), elbow flexors CSA (cm^2^)	Higher increases in trained arm strength for leg-BFR group (2.5 ± 0.4 kg vs 0.8 ± 0.4 kg) No effects on muscle CSA Untrained arm increased strength in both the leg-BFR group (0.8 ± 0.8 kg) and no-BFR group (1.5 ± 1.1 kg)	Vertical strength transfer facilitated for the targeted UB muscles trained before leg resistance sessions that supposedly produced systemic factors Vertical strength transfer noted for the untrained upper limb after leg resistance sessions
Cook et al. [54] (2014)	Semiprofessional male rugby players	20	21.5 ± 1.4	184 ± 5.0	95.6 ± 10.4	3 weeks, 3 × /week	SG with leg BFR vs SG without leg BFR	1-RM bench press (kg) Testosterone (pg/mL), cortisol (ng/mL)	Significantly greater improvements in bench press for leg BFR group (5.4 ± 2.6 vs 3.3 ± 1.4 kg; *p* = 0.0044, 1.4% ± 0.8%) Significant associations between mean acute salivary testosterone response and bench press strength (*r* = 0,45, *p* = 0,0233)	Vertical strength transfer facilitated for the targeted UB muscles trained before leg resistance sessions that produced systemic factors
Ampomah et al. [43] (2019)	Adults with low back pain	32	28.4 ± 9.2	All-limb BFR training followed by trunk extension group: 168.5 ± 8.8 Normal training followed by trunk extension group: 170.1 ± 9.8	All-limb BFR training followed by trunk extension group: 75.7 ± 16.7 Normal training followed by trunk extension group: 71.4 ± 14.4	10 weeks, 2 × /week	All-limb BFR resistance exercise followed by trunk extension group vs normal resistance exercise followed by trunk extension group	Trunk extensor MVIC (Nm) and endurance (s), erector spinae CSA (cm^2^)	No significant changes in erector spinae strength, endurance, or CSA	No vertical strength transfer facilitated for the targeted UB muscle when all limbs were trained with light BFR approach
West et al. [49] (2010)	Recreationally active male individuals	12	21.8 ± 1.2	178 ± 2.0	74.1 ± 3.3	15 weeks, 3 × in 2 weeks for each arm until 7th week, then 2 × /week each arm until 15th week	One group with two different protocols on separate days: unilateral arm strength training alone vs contralateral arm strength training followed by high-volume leg resistance exercise	Elbow flexors MVIC (Nm), 1-RM & 10-RM elbow flexion (kg) Testosterone (nM), GH (μM), IGF-1 (nM)	Significantly elevated endogenous hormones after leg trainings (*p* < 0.001) did not result in an increased arm hypertrophy response or strength after the programme	No vertical strength transfer facilitated for the targeted UB muscles trained before leg resistance session that produced systemic factors
Rønnestad et al. [18] (2011)	Untrained male individuals	9	20–34	181 ± 3.0	79 ± 3.0	11 weeks, 2 × /week each arm	One group with two different protocols on separate days: unilateral arm strength training alone vs contralateral arm strength training preceded by high-volume leg resistance exercise	1-RM elbow flexion (kg), elbow flexors CSA (cm^2^) Testosterone (nM), GH (mlE/l), cortisol (mlE/l)	Strength improvements higher after leg training (21%) than without (14%) Significant increases in the trained arm’s largest area after leg training (*p* < 0.001) Significantly increased serum testosterone and GH levels after leg training (*p* < 0.05)	Vertical strength transfer facilitated for the targeted UB muscles trained after leg resistance sessions that produced systemic factors
Jakobsson et al. [44] (2021)	Healthy male and female individuals	M: 9 W: 8	30.9 ± 7.6	High resistance-lower volume leg training before UB training group: 170.2 ± 10.1 High volume-lower resistance leg training before UB training group: 173.7 ± 3.9	High resistance-lower volume leg training before UB training group: 68.1 ± 12.0 High volume-lower resistance leg training before UB training group: 74.9 ± 8.0	10 weeks, 3 × /week	Higher resistance-lower volume LB exercise before UB strength training group vs higher volume-lower resistance LB exercise before UB strength training group	1-RM bench press (kg), lat pull-down (kg) Testosterone (nmol/l), GH (ug/l)	No significant differences in UB strength improvements Greater increase in GH for high-volume group	No vertical strength transfer facilitated for the targeted UB muscles trained after leg resistance sessions that elevated GH levels
Bartolomei et al. [21] (2018)	Trained male individuals	20	24.9 ± 2.9	High resistance-lower volume leg training after UB training group: 177.0 ± 5.6 High volume-lower resistance leg training after UB training group: 177.54 ± 5.9	High resistance-lower volume leg training after UB training group: 88.7 ± 17.2 High volume-lower resistance leg training after UB training group: 82.8 ± 9.1	6 weeks, 4 × /week	Higher resistance-lower volume LB exercise after UB strength training group vs higher volume-lower resistance LB exercise after UB strength training group	1-RM bench press (kg), 50% of 1-RM ballistic bench press (W), arm muscle size (cm)	More significant hypertrophy for arm muscle area (*p* = 0.046), more significant UB strength increase (*p* = 0.007), and more significant UB power expression (*p* = 0.011) with high volume leg training approach	Vertical strength transfer facilitated for the targeted UB muscles trained before leg resistance sessions that supposedly produced systemic factors
West et al. [48] (2009)	Recreationally active male individuals	8	20.0 ± 1.1	179 ± 3.0	84.1 ± 4.1	Acute study protocol	One group with two different protocols on separate days: unilateral arm strength training alone vs contralateral arm strength training followed by high-volume leg resistance exercise	Essential amino acid and branched-chain amino acid levels in biceps brachii (μM) Testosterone (nM), GH (μM), IGF-1 (nM)	Provoked anabolic hormone response by leg training (testosterone, *p* < 0.001; GH, *p* < 0.001; IGF-1, *p* < 0.05) did not influence arm-muscle protein synthesis rates	No enhanced protein synthesis for the targeted UB muscles trained before leg resistance session that produced systemic factors
Spiering et al. [57] (2009)	Healthy male individuals	6	26 ± 4	176 ± 5	75.8 ± 11.4 kg	Acute study protocol	One group with two different protocols on separate days: bilateral LB strength training after high-volume UB resistance exercise vs bilateral LB strength training alone	Testosterone (nM/l), muscle androgen receptors (au)	Significantly increased serum testosterone after UB training vs leg training only (*p* = 0.01–0.04; effect size = 0.63–1.03). Testosterone area under the curve 14% greater (*p* < 0.01; effect size = 0.60)	Vertical strength transfer potentially reflected in beneficial systemic responses for the targeted LB muscles trained after UB resistance session
Spillane et al. [58] (2015)	Trained male individuals	8	23.62 ± 4.86	179.07 ± 5.34	96.69 ± 15.27	Acute study protocol	One group with two different protocols on separate days: bilateral LB strength training after high-volume UB resistance exercise vs bilateral LB strength training alone	Testosterone (nM/l), β-Catenin (Abs/ug)	No significant difference in testosterone levels between the groups. β-Catenin significantly greater after UB training vs leg training only at 3 h (*p* = 0.001) and 24 h (*p* = 0.001) post-exercise	Vertical strength transfer potentially reflected in beneficial systemic responses for the targeted LB muscles trained after UB resistance session (with no reference to testosterone levels)

*BFR* blood-flow restriction, *CG* control group, *CSA* cross-sectional area, *GH* growth hormone, *IGF-1* insulin-like growth factor-1, *MVIC* maximal voluntary isometric contraction, *UB* upper body, *LB* lower body, *RM* repetition maximum, *SG* whole-body strength training group

**Table 3:** Vertical strength transfer effect with the exercise of one body segment

Study	Participant characteristics					Training protocol			Key findings	Vertical strength transfer
	Population	*n*	Age (years)	Stature (cm)	Body mass (kg)	Duration	Intervention	Outcome measures of interest		
Kraemer et al. [45] (2004)	Untrained physically active females	85	23.1 ± 3.5	SG (3–8 RM: 163.7 ± 7.5; 8–12 RM: 165.4 ± 5.4) UB- only training groups (3–8 RM: 165.5 ± 7.2; 8–12 RM: 166.7 ± 6.2) Control group: 164.5 ± 6.2	SG (3–8 RM: 64.1 ± 8.8; 8–12 RM: 63.2 ± 7.0) UB- only training groups (3–8 RM: 66.7 ± 10.7; 8–12 RM: 65.7 ± 12.1) Control group: 65.9 ± 11.2	24 weeks, 3 × /week	SG vs UB-only training groups vs CG	1-RM squat (kg), 30%/60%/90% of 1-RM jump squat (W)	Small trend for improvement in LB strength for 3–8 RM and 8–12 RM UB-only groups (*p* = 0.08 and *p* = 0.10, respectively) LB power not significantly influenced in UB-only groups	Non-significant vertical strength transfer noted from UB strength training to LB strength or power improvement
Othman et al. [50] (2018)	Boys	48	10–13	High load-low rep unilateral leg training group: 147.5 ± 5.5 Low load-high rep unilateral leg training group: 147.5 ± 4.7 Control group: 146 ± 3.5	High load-low rep unilateral leg training group: 40.1 ± 3 Low load-high rep unilateral leg training group: 38.3 ± 3.5 Control group: 38.8 ± 8.1	8 weeks, 2 × /week	High load-low repetition unilateral LSG vs low load-high repetition unilateral LSG vs CG	Elbow flexors MVIC (N), handgrip strength (N)	Untrained arm strength increased more in high load-low rep group for elbow flexor MVIC (14.1% vs 3.3%; *p* < 0.0001) and handgrip (16.1% vs 7.5%)	Vertical strength transfer noted for the untrained UB muscles after high-resistance LB exercise sessions
Chaouachi et al. [51] (2019)	Boys	48	11.9 ± 6 1.2	High load-low rep unilateral leg training group: 147.5 ± 5.5 Low load-high rep unilateral leg training group: 147.5 ± 4.7 Control group: 146 ± 3.5	High load-low rep unilateral leg training group: 40.1 ± 3 Low load-high rep unilateral leg training group: 38.3 ± 3.5 Control group: 38.8 ± 8.1	4 weeks of detraining	High load-low repetition unilateral LSG vs low load-high repetition unilateral LSG vs CG	Elbow flexors MVIC (N), handgrip strength (N)	Non-significant upper limb strength deficits after the detraining period	Retention of strength gained in the untrained UB muscles after the LB resistance exercise plan
Pietrangelo et al. [52] (2019)	Older males	31	71.77 ± 4.06	167 ± 8	80.47 ± 12.09	12 weeks, 3 × /week	LSG vs EG vs CG	Handgrip strength (kg)	Significant strength increases in the upper limbs for both LSG and EG (*p* = 0.061 and *p* < 0.001 respectively)	Vertical strength transfer noted for the untrained UB muscles after LB resistance and cardiorespiratory endurance trainings that supposedly produced systemic factors
Ceci et al. [31] (2020)	Older adults	32	70.73 ± 3.98	DNR	DNR	12 weeks, 3 × /week	LSG vs EG vs leg-neuromuscular electrical stimulation training group	Handgrip strength (N) Superoxide dismutase (U/mL)	Increased superoxide dismutase activity and handgrip strength in EG	Vertical strength transfer noted for the untrained UB muscles after LB cardiorespiratory endurance trainings that produced systemic factors
Magdi et al. [22] (2021)	Trained males & trained females	M: 40 W: 29	20.2 ± 2.2	Men 178.9 ± 5.7 Women 165.1 ± 5.6	Men 76.1 ± 7.8 Women 60.2 ± 7.1	10 weeks, 2 × /week	Unilateral leg accentuated eccentric loading training group vs CG	Ipsilateral elbow flexor MVIC (kg), 1-RM strength (kg), and muscle power (W)	Strong ipsilateral lower-to-upper limb cross-transfer effect with significantly increased MVIC in ipsilateral arm (men: 14.7%, women: 69.4%), strength with 1-RM (men: 10.5%, women: 20.6%), and power at low (men: 59.0%, women: 72.6%), medium (men: 47.1%, women: 60.8%), and high loads (men: 19.6%, women: 53.3%) Mass gain not observed and no significant differences in the magnitude of strength change between sexes	Vertical strength transfer noted for the untrained UB muscles after unilateral eccentric leg resistance trainings
Aman et al. [23] (2021)	Females	34	56.05 ± 5.21	Leg massed rehabilitative training programme group: 155 ± 4.19 Leg distributed rehabilitative training programme group: 156 ± 6.04 CG: 154 ± 6.07	Leg massed rehabilitative training programme group: 67.43 ± 7.64 (before), 66.71 ± 8.15 (after) Leg distributed rehabilitative training programme group: 65.48 ± 9.17 (before), 64.92 ± 9.23 (after) CG: 68.17 ± 5.55 (before), 69.25 ± 5.45 (after)	12 weeks, 3 × /week; 5 × /week, massed group: rest-to-practice ratio 1:1; distributed group: rest-to-practice ratio 2:1	Leg massed rehabilitative training programme group (concentrated within shorter time) vs leg distributed rehabilitative training programme group (concentrated around longer time) vs CG	Elbow flexors MVIC (N)	Significantly improved level of strength in the untrained arms for both groups (distributed practice right hand: 58.3%, left hand 44%; massed practice left hand: 33.9%), with significantly higher impact by distributed practice (in right hand: 45.1%, in left hand 33.4%)	Vertical strength transfer noted for the untrained UB muscles after LB resistance trainings (especially after distributed practice)

*CG* control group*, DNR* did not report, *EG* cardiorespiratory endurance training group, *LB* lower body, *LSG* leg strength training group*, MVIC* maximal voluntary isometric contraction, *RM* repetition maximum, *SG* whole-body strength training group, *UB* upper body

## Results

### Search Results

**Fig. 1:**
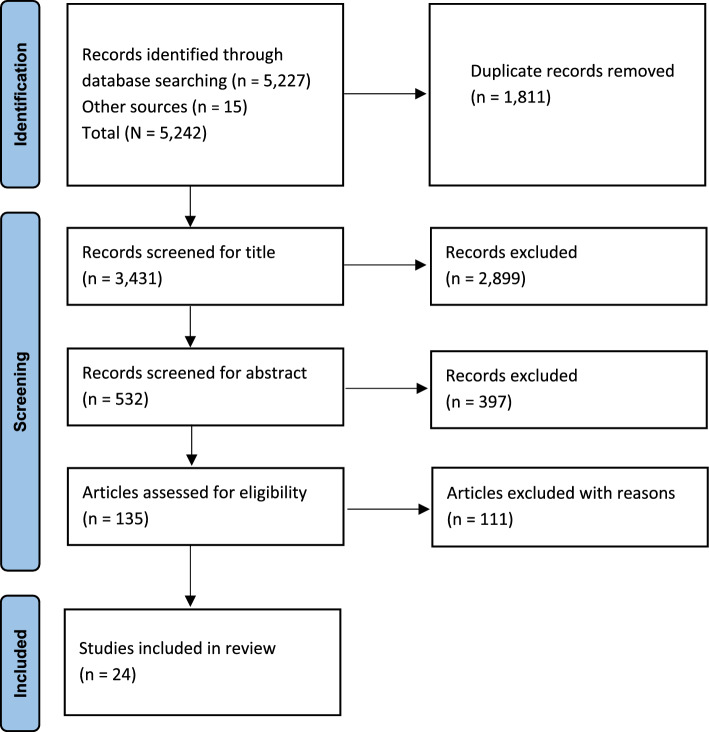
PRISMA (Preferred Reporting Items for Systematic Reviews and Meta-Analyses) flowchart illustrating the step-by-step process leading to identification of studies eligible for the review

Initial database searches resulted in the identification of 5227 articles with 15 additional articles identified through other sources. Following the removal of duplicates (*n* = 1811) and irrelevant articles (*n* = 2899), 532 articles were retained for the abstract screening process. Abstract screening resulted in the exclusion of 397 articles, leaving 135 full-text articles to be assessed for eligibility. A further 111 articles were excluded because of not meeting inclusion/exclusion criteria, resulting in 24 articles being included in the scoping review (Fig. 1).

Four studies included mixed-sex participants [22, 31, 43, 44], two included only female individuals [23, 45] and 18 involved only male individuals [16–18, 21, 24, 38, 46–55]. The age range was 10–73 years inclusive of all eligible studies. However, children and older adults were present in only six [16, 31, 50–52, 55] out of 24 studies included in the review, which significantly expanded the age range. Out of 24 studies, 4 were acute study protocols [17, 48, 56, 57], 1 examining UB resistance and a LB endurance exercise session [17], and other 3 exploring UB resistance and a LB resistance exercise session [48, 56, 57]. Notably, only one study from this review involved an athletic population [54].

### Upper Body Resistance Exercise with Lower Body Endurance Exercise

Table 1 provides a summary of the characteristics, outcome measures and key findings of the eligible studies that investigated VST when UB resistance exercise was added to an endurance exercise session. One study paired UB exercise with running [38], while three studies paired UB exercise with cycling [16, 17, 55]. Verney et al. [55] reported a 9–13% increase in maximal isometric and isokinetic torque of the knee extensors after concurrent 3 × 12 min cycling bouts and UB strength training across 14 weeks. This study [55] involved active older adults, who followed a progressive resistance training programme for upper limbs and trunk muscles. The participants performed three exercises per UB muscle group with three sets for each muscle every session [55]. Heavier weights were used for shoulder and chest exercises [range from 10–12 to 3–6 of repetition maximum (RM)], while exercises for arms initially had lighter resistance (20-RM), and later progressed to heavier resistance (10–12-RM) [55]. Abdominal and lower back exercises used only bodyweight throughout the programme with three sets of 20 repetitions [55]. Another study that tested LB strength [38] involved active young male individuals who failed to make any improvements on 1-RM leg extension and 1-RM squat tests after a 12-week training plan. This plan involved 40-min continuous running protocols (2 × /week) and 200–800-m interval running bouts (2 × /week), both paired with high-intensity UB exercises (10-RM and 5-RM load progression) on the same days [38]. Importantly, however, the Wingate leg cycling test showed that the group with added UB strength sessions increased both the peak (39 W) and mean leg power (20 W), whereas the running-only group experienced a decrease with these two measures (peak power − 8 W, mean power − 14 W) [38].

Two of the included studies examined leg muscle fibre adaptations to this training configuration [16, 38]. The intervention by Kraemer et al. [38] observed the preservation of type I and type IIc fibre areas of the vastus lateralis with the inclusion of UB strength trainings compared with the members of a running-only group who saw a significant decrease [38]. Another study [16], being a follow-up to the previous one [55], evidenced a 73% increase of the satellite cell number per type II fibre of the cycling-trained vastus lateralis (*p* = 0.04), aided by significant increases of type IIa fibre areas (13%, *p* = 0.03) when UB strength exercises were joined to cycling sessions [16]. Furthermore, in a study by Moberg et al. [17], various circulating endocrine factors were reported by the combination of an intensive endurance-based cycling protocol (5 × 4 min high intensity interspersed with 25-min low intensity) and subsequently exercised triceps muscle. These systemic exerkines favourably alter muscle tissues in both body regions leading to enhanced oxidative and glycolytic adaptations [17], with a potential to increase muscle mass and strength in both areas [58].

### Upper Body Resistance Exercise with Lower Body Resistance Exercise

Table 2 provides a summary of the characteristics, outcome measures and key findings of the eligible studies that investigated VST facilitation with the combination of UB resistance training and LB resistance training in the same session. Fourteen studies were identified [18, 21, 24, 43–49, 53, 54, 56, 57]. Twelve of them investigated how a LB resistance exercise session facilitated an UB strength session [18, 21, 24, 43–49, 53, 54]. This resulted in an augmented increase of UB strength or power in seven studies [18, 21, 24, 45–47, 54], while no enhancement was found for UB strength in four studies [43, 44, 49, 53], nor for acute arm muscle protein synthesis in one study [48]. Although no chronic training interventions were identified with a reverse facilitating order (UB for LB), there were two studies that examined how UB resistance exercise may facilitate LB strength training adaptations via immediate systemic responses [56, 57]. In one protocol, muscle androgen receptor content from the vastus lateralis was preserved with the preceding high-volume UB exercise session [56], while the same protocol from another study [57] revealed beneficial binding of the vastus androgen receptors to the DNA and increased canonical Wnt/β-catenin signalling [57], suggesting the potential for a strength-enhancing effect of a targeted leg muscle when it was exercised after resistance training of UB muscles.

Kraemer et al. [45] reported increased UB power measured by ballistic bench press and greater arm muscle hypertrophy for the group with added leg strength training sessions to UB strength training sessions compared with the UB-only exercise group. In contrast, Walker et al. [53] did not observe any additional improvement in arm muscle strength when other muscle groups were jointly trained within the same sessions. Notably, plasma insulin-like growth factor-1 did not change, while myostatin levels were equally lowered with arm-only exercise and whole-body exercise [53]. Furthermore, when unilateral arm resistance training was followed by endocrine-producing leg resistance training, arm strength improvement was enhanced for both untrained [24, 46] and trained [47] young male individuals. Two of these studies [46, 47] incorporated blood-flow restriction (BFR) leg exercises, which led to the augmented strength responses in the trained arm [46, 47]. Similarly, the only study that involved an athletic population [54] observed facilitated improvements in a 1-RM bench press when UB muscles were trained alongside leg-BFR exercises after only 3 weeks [54]. Contrary to these findings, BFR applied to all limbs did not augment trunk extension strength when trunk muscles were trained alongside arms and legs [43]. The participants from this study [43] were untrained adults with back pain who followed a light 10-week training programme with three sets of leg extension, calf raises and arm curls at 25% of their maximal voluntary isometric contraction (MVIC) two times a week [43].

Studies with a within-subject design showed conflicting findings when male participants trained different arms on different occasions [18, 49]. Acutely elevated hormonal milieu by a leg resistance session failed to enhance the protein synthesis rate in previously trained elbow flexors of a trained arm [48], and this exercise protocol did not support unilateral arm strength improvement after a 15-week exercise plan [49]. However, when the LB region was trained prior to a single arm, both strength and hypertrophy were greater in this arm following an 11-week training plan [18]. Finally, two studies investigated whether performing high-volume leg exercise sessions could enhance the effectiveness of UB strength training sessions targeting multiple muscle groups (chest, back, shoulders and upper limbs) [21, 44]. A study with 17 mixed-sex participants found no enhancement for UB strength when UB exercises followed LB sessions despite produced elevations in growth hormone levels [44]. In contrast, a study with 20 trained male individuals, who exercised their UB muscles before high-volume LB sessions, reported more significant hypertrophy for the arm muscle area, more significant bench press improvement, and more significant UB power expression compared with the low-volume leg exercise group [21].

### Vertical Strength Transfer with the Exercise of One Body Segment

Table 3 provides a summary of the characteristics, outcome measures and key findings of the eligible studies that investigated VST with the exercise of one body segment. Six studies [22, 23, 31, 50–52] of the seven included [22, 23, 31, 45, 50–52] evaluated changes in untrained UB strength as an outcome of a LB exercise intervention. All the interventions reported increased strength of upper extremities measured by handgrip and MVIC after various exercise plans including: bilateral LB strength exercise [23, 50, 51], unilateral LB strength exercise [22] and cardiorespiratory endurance LB exercise [31, 52]. Only one investigation examined the influence of an UB strength training intervention for the untrained LB strength [45]. The authors reported a marginal strength improvement on a 1-RM squat for both the 3–8 RM training group (*p* = 0.08) and 8–12 RM training group (*p* = 0.10) [45].

Ben Othman et al. [50] reported a higher strength increase in untrained upper extremities measured by elbow flexor’s MVIC and handgrip for a high load-low repetition group of boys compared with the low load-high repetition group. These strength improvements were retained after 4 weeks of a detraining period for the same participants [51]. Furthermore, Pietrangelo et al. [52] and Ceci et al. [31] reported improved handgrip strength in older male individuals as a result of a leg resistance [52] and leg endurance training programme [31, 52]. Aman et al. [23] also demonstrated a significantly improved level of strength in the untrained arms for middle-aged female individuals allocated to groups with distributed rehabilitative practice and massed rehabilitative practice [23]. Finally, Magdi et al. [22] organised unilateral leg training with accentuated eccentric loading for the group of trained young male and female individuals, examining the effect on the ipsilateral arm. There was a significant increase in arm MVIC and power at low, medium, and high loads, with no gains in muscle mass nor differences between the sexes compared to the control group after a 10-week plan [22].

## Discussion

The purpose of this systematic scoping review was to investigate the existence of the VST phenomenon as a response to various UB and LB exercise configurations and to identify potential mechanisms underpinning its occurrence. The main findings are as follows: (1) concurrent UB strength and LB endurance training (i.e. running or cycling) for older adults may stimulate beneficial satellite cell expression in both regions, benefitting LB muscle morphology and LB strength enhancement. This training combination also shows the potential to protect leg muscle fibres from a catabolic environment and to preserve power-generating capacity in LB muscles via neurophysiological mechanisms, highlighting promising applications for athletic populations, (2) high-volume or BFR type of LB resistance exercise may facilitate strength improvements in previously or subsequently trained upper limbs, and previously or simultaneously trained multiple UB muscle groups. The opposite direction of transfer (i.e. UB to LB) has not been investigated with strength testing outcomes, (3) high-volume UB resistance exercise beneficially modulates androgen receptor response in subsequently exercised quadriceps, demonstrating the potential for a strength-enhancing effect of LB muscles, (4) strength training for LB muscles may result in improved strength of untrained upper extremities, and (5) VST likely occurs because of the combination of neurological adjustments and circulating endocrine factors. Future research should examine the extent of VST facilitation in an athletic population, especially with respect to the influence of UB resistance training on LB strength or power-generating adaptations.

### Upper Body Resistance Exercise with Lower Body Endurance Exercise

An important finding from this review that has potential implications for athletic performance is the noted protective role of UB strength training for the prevention of leg power loss and type I and type IIc leg muscle fibre area when it was performed after various running sessions across a 12-week training period [38]. This finding is reinforced with the observed increase of type II fibre area and satellite cell number per type II fibre in the vastus lateralis [16], as well as improved strength of the quadriceps muscle [55] when UB exercise was joined to the leg cycling sessions for older adults [16, 55]. In addition, when high-intensity interval cycling for endurance was performed prior to arm resistance training, this led to the production of circulating factors that beneficially modulate muscle adaptations in both body regions, potentially leading to strength improvements on a whole-body level [17].

A possible explanation for the leg-protective effect by concurrent UB strength training and LB endurance training may lie in various physiological mechanisms provoked by the exercise of an entire UB region [38]. 5′Adenosine monophosphate-activated protein kinase (AMPK) is well known for the regulation of energy homeostasis [59], and it responds to endurance training [60], acting as a suppressor of the mammalian target of rapamycin (mTOR) pathway known for its anabolic effects [61–63]. However, when resistance exercise follows endurance training, mTOR1 signalling is not inhibited despite the pre-activation of AMPK [64], and anabolic gains are not compromised [65]. This could explain why UB strength improvement was not negatively impacted when these muscles were trained after the running sessions [38]. In reverse order of signalling pathways, anabolic mediators such as mTOR1, insulin-like growth factor-1 and serine-threonine protein kinase (Akt) after resistance exercise do seem to suppress catabolic processes in human organisms [66–69]. This “switch’’ between different pathways affects muscle fibre size depending on the dominant type of activity [70, 71]. Thus, UB strength training may have systemically induced endocrine factors that interfered with LB muscle morphology in the mentioned protocols [72]. For instance, one study observed that performing an UB strength training 1 day after a muscle-damaging leg exercise session accelerated the recovery of concentric force generation in the trained leg [73], which may be explained by the recovery potential of circulating anabolic hormones such as testosterone [74]. Notably, Kraemer et al. [38] organised a training plan with multi-joint exercises for large muscle groups (i.e. bench press, military press, latissimus pull-down), with the capacity to stimulate testosterone production for systemic influence [56]. This circulating androgen might have improved nerve conduction velocity and myelination [75, 76], potentially leading to faster and stronger electrical signals to any muscle in the body. Indeed, strength training intensifies CNS for increased impulses [77], leading to modified behaviour of the motoneurons [78], which could have resulted in the protective effect on the leg power output [38]. Moreover, two acute exercise studies have shown that UB strength training could effectively potentiate quadriceps’ androgen receptor response to exercise [56, 57]. This potentiation may offer protection against catabolism [56] aiding the anabolic enhancement of strength and power capacity in the targeted muscles [75]. Beyond the effects of testosterone, muscle growth and maintenance are also influenced by other complex regulatory mechanisms. One such systemic factor is follistatin, a glycoprotein induced by resistance exercise [28, 79, 80]. Elevated follistatin induces muscle hypertrophy [28] and may decrease myostatin levels [15], a myokine that negatively affects muscle mass [81]. One study from this review observed lowered total myostatin levels by 20% with as little as three exercise sets for elbow flexors in the training plan [53]. Hence, intensive exercise of an entire UB segment may have the potential to produce endocrine factors that could counterbalance leg muscle fibre atrophy seen after long running sessions [39], resulting in the preservation of high-intensity performance for the LB [38]. This could bring novel considerations for exercise sequence programming that needs to address both aerobic power and high-intensity force production by lower extremities for an athletic population competing in multi-directional sports [82, 83]. Future research should explore this link further as it may have significant implications in sports like soccer where current UB resistance sessions do not seem to result in pronounced neurophysiological adaptations [84] compared to the requirements placed on the LB musculature [85, 86].

Further evidence for beneficial effects of combining LB endurance and UB strength exercise in one session arrived from Moberg et al. [17], who found that elevations in PGC-co-activator-γ-1 (PGC-1α)1 and PGC-1α4 were markedly larger when arm resistance training followed after the endurance-based high-intensity interval cycling session. PGC-1α1 is elevated after endurance training [87, 88] and has a beneficial role in oxidative adaptations, promoting fatigue resistance [87, 89], while PGC-1α4 expression is greater after resistance exercise, and has a facilitating role for muscle hypertrophy [58] and glucose uptake via augmentation of key glycolytic genes [90]. These two isoforms are a significant part of a large PGC-1α transcription coactivator group that serves as a key stimulator of mitochondrial biogenesis heavily linked to lactate metabolism [91], a beneficial modulator of metabolic genes [92, 93] and an efficient preserver of muscle mass in the face of a catabolic environment [94] with a role on a whole-body level [95]. Concurrent exercise modes with the same muscles (e.g. cycling and leg resistance exercise) have been shown to increase these two isoforms [65, 96], but here the systemic effect was noted with separate muscles (legs and triceps). A similar occurrence happened in a study by Birnbaumer et al. [30] who reported elevated systemic blood lactate levels after the warm-up activity that involved 30-s all-out running, which improved performance in the subsequent pull-up exercise via enhanced glycolytic and oxidative metabolism [30]. This cross-tissue lactate utilisation from concurrent types of exercise may explain the augmentative effect on exercise performance [91, 97–99]. Nevertheless, the evidence for long-term benefits is still scarce and requires careful investigations with chronic exercise adaptations in the future.

Two intervention studies from this review examined the cross-effects of concurrent UB strength training and LB cycling sessions spread across a 14-week period for older adults [16, 55]. With the absence of control group, it remains unclear whether the improvement in leg strength [55], followed by a beneficial similar increase in the satellite cells per fibre in both regions [16], was supported by the physiological cross-talk [17]. Although an older population could increase strength solely on the basis of endurance exercise [100], concurrent exercise modes lead to augmented strength improvements [101, 102], optimising both cardiovascular and neuromuscular gains [102]. Therefore, combining LB endurance training with UB resistance training may provide endurance-related adaptations to resistance-trained muscles and vice versa. Future research should further explore this effect as it may hold significance for the population with the limited training capacity like older adults who could obtain health benefits from two distinct exercise types in one training session.

### Upper Body Resistance Exercise with Lower Body Resistance Exercise

This review has identified that neurophysiological adaptations arising from the exercise of one body region may augment strength development in another body region [18, 24, 45, 46, 54]. However, VST is less likely to be facilitated if multiple factors are not accounted for, such as the exercise load and volume [43, 53], exercise order [48, 49] and exercise type [44].

#### Neurophysiological Underpinning of the Facilitated Transfer

Training of the whole body elicits greater systemic responses compared with the protocols that involve a smaller number of muscle groups [15, 103]. In addition to anabolic hormones [74], these signals also involve myokines that regulate muscle adaptations such as myostatin and follistatin [15, 79, 81], modified satellite cell activity [104] and triggered anabolic pathways like Akt/mTOR [62, 68]. While exercise sessions that involve large muscle groups, being high in volume and moderate to high in intensity, are critical to producing substantial endocrine responses [74, 103, 105–108], a weak or insufficient stimulus by the selection and intensity of included exercises may lack the capacity to affect these changes [109]. This could be seen in the studies that applied LB resistance exercises with a high-volume approach, which often resulted in an augmented increase of concurrently trained arm muscles [18, 43], whereas low-volume protocols showed conflicting results [43, 54]. When both UB and LB regions are trained together, it is difficult to distinguish whether physiological or neurological adaptations facilitate the VST. For example, UB power (measured by a ballistic bench press) improved significantly more after a whole-body strength training programme than after the same UB exercise plan without LB involvement (delta change + 68 W, *p* < 0.05) [45]. This may have occurred as a result of magnified neuromuscular adaptations arriving from heavy loads with the enhanced neural drive for UB muscles by the inclusion of intensive LB contractions [2, 110–112]. Hence, it looks likely that both neurological and physiological mechanisms underpin the adaptations that lead to the VST occurrence when UB and LB muscles are concurrently trained.

Three studies from this review demonstrated a higher increase in UB strength when UB exercises were performed alongside leg-BFR exercises compared with the leg exercises without BFR [46, 47, 54]. This probably occurred via emphasised circulating endocrine factors [12], as evidenced in the two studies that took the measures [46, 54]. Likely mediators of these physiological cross-adaptations are lactates [30], which accumulate under hypoxic conditions caused by BFR [113] or by high-volume exercise protocols [114–116]. Lactates have the ability to impact distant tissues as signalling molecules [91, 98, 99, 117], stimulate reactive oxygen species production [118], increase type II fibre recruitment [119, 120], and elevate anabolic hormone levels [113, 121, 122], promoting hypertrophic effects [113, 123]. In addition, lactate produced by one muscle can be systemically utilised as an effective energy fuel by other “recipient” muscles [97]. Its shuttle transport is supported by elevated testosterone [172], a hormone proposed as a possible facilitator of the VST in four studies [18, 24, 46, 54]. While oxidative muscle fibres use lactates directly [97], type II fibres primarily dispose them via gluconeogenesis [125]. Lactates are also preferably used as a source of energy in brain cells [25, 126], where they promote neuroplasticity and cerebrovascular plasticity [25, 26], with the potential to enhance corticospinal excitability and reduce intracortical inhibition [127]. This mechanism might explain significant strength increases of the contralateral (untrained) arm after leg-supported unilateral arm training [24, 47], which points to an improved motor unit recruitment in that limb [105, 128].

In addition to the lactate-induced neuroplasticity, testosterone has also been shown to significantly associate with neuromuscular performance measured by squat jump and change-of-direction speed in young athletes [129], as well as with dose-dependent and concentration-dependent increases in maximal voluntary leg strength and leg power in healthy young men [27]. While suppression of endogenous testosterone production diminishes strength gains [130], its elevation leads to increased levels of released neurotransmitters and reorganisation of neurons [75]. Therefore, the inclusion of exercises from a distant body region might have had a vital neurophysiological complementary influence on another region’s strength training adaptations with the production of circulating lactates and androgens. When considering practical applications for athletic populations, however, it is important to recognise that, with the exception of Cook et al. [54], who studied semi-professional rugby players, all other investigations involved untrained or recreationally active individuals who could gain notable improvements in strength and hypertrophy with a smaller number of resistance training sessions [131]. Consequently, the extent to which these cross-training adaptations are applicable and impactful for a sports population remains unclear. Nonetheless, presented findings could hold potential significance for leg-dominant athletes such as soccer players who do not seem to prioritise strength development in UB muscles [84]. Future research is, therefore, warranted to investigate if this population may benefit from emphasising high-resistance UB exercises in addition to LB sessions, optimising overall athletic performance via increased neurophysiological effects.

#### High-Volume Resistance Exercise Facilitation for the Isolated Muscle from a Remote Body Region

In this review, three studies showed that the anabolic-producing type of weight training for LB muscles had a positive impact on strength development for the elbow flexors that were trained earlier in the session [24, 46, 47], likely due to augmented neurophysiological adaptations [47]. In contrast, a similar approach by West et al. [49] did not result in any enhancements of arm strength improvement after the intervention. This finding [49] is in line with research showing that raised levels of endogenous anabolic hormones do not always correlate with hypertrophy and strength gains [132–134], questioning their role in the facilitation of strength [132]. Importantly, however, this within-subject study [49] required participants to ingest 18 g of protein before and after each of the workouts that were separated by 24 h, involving a unilateral arm exercise session followed by a high-volume LB exercise session first, and a contralateral arm exercise session alone the next day. After the combined session, post-exercise protein availability may have been prioritised for consumption by the leg muscle cells [135], whereas the arm-only training protocol had the same amount of protein content readily available to the exercised arm muscle [49]. Furthermore, with the increased amino acid presence in 2 consecutive days, training of the contralateral arm may have benefitted from the previous day’s high-volume session via prolonged anabolic pathway signalling [136] and altered systemic metabolic state potentiating muscle stem cells [72] in the untrained arm. For example, muscle damage in one limb triggers metabolite signals that prime distant stem cells in the opposite limb [72], placing them in a prolonged mTOR-dependent “alert state” ready for potential future modifications if required [137].

The opposite exercise design, in which anabolic-producing weight training from one body region was positioned before the strength training of a muscle from a remote body region, proved effective in augmenting a targeted muscle’s response [18, 56, 57], possibly due to the contractions occurring under an altered systemic environment with the additive effect [18, 30, 56, 57]. For instance, weight training for several UB muscles (i.e. chest, back, shoulders, arms) was efficient in potentiating favourable transcriptional changes in the DNA-ribosome complex of a subsequently exercised quadriceps [56, 57], revealing the potential for strength enhancement of LB muscles [138–141], though without a clear link to the elevated testosterone [57]. By reversing the direction of facilitation, LB resistance training sessions conducted before isolated biceps exercises provided a considerable boost in elbow flexor strength after the training programme [18]. Therefore, using the logic that elevated systemic factors may remodel muscles trained later in the session [56, 74], likely via complementing neurophysiological adaptations [75, 124], it looks conceivable to suggest that high-volume exercise of the LB musculature may provide support for the progress of arm strength trained afterwards, which could be critical during the rehabilitation process after injury. As an example, this concept might also be applied to an isolated hamstring exercise placed after the UB resistance session to maximise its strength gains, potentially leading to enhanced athletic performance [142, 143] or a hamstring injury risk reduction [144]. It remains unclear, however, whether this training combination has the capacity to result in significant strength improvement for an athletic population, which should be further investigated.

#### High-Volume Resistance Exercise Facilitation for Multiple Muscles from a Remote Body Region

The previous section showed that when the goal was to maximise strength exercise response of a single muscle (e.g. biceps brachii), high-volume exercise of other body region was partly successful when positioned afterwards [24, 47–49], but reliably effective when positioned before [18, 56, 57]. In contrast, however, when the goal was to augment strength gains for multiple muscles across the whole UB region, preceding high-volume LB exercise failed to support it [44], whereas succeeding high-volume LB exercise successfully facilitated it [21]. The explanation for this discrepancy may lie in the impaired contractions from large UB muscle groups when they were trained later in the session due to the reduction in voluntary muscle activation [145] resulting from the previous leg workout. The central fatigue [146] may not substantially affect contractions of a single muscle [18], but it might hinder the activation of multiple muscles from various UB areas and result in the lack of strength enhancement [44]. Furthermore, a study that failed to facilitate strength adaptations [44] had female individuals for half of the participants compared to the study that involved only male individuals and proved successful [21]. The differences between the sexes might have affected physiological responses to exercise (e.g. no difference in testosterone was noted between the female training groups) [147], contributing to the deficiency of a transfer effect [44].

The inconsistency in findings between West et al. [49] (no strength facilitation for previously exercised biceps muscle) and Bartolomei et al. [21] (successful strength facilitation for previously exercised multiple UB muscles) may originate from the absence of local testosterone production following the exercise of a singular muscle from the UB [48, 49] compared to the comprehensive engagement of a total UB segment [56, 57]. This broader engagement likely triggered an anabolic response in the targeted UB [56], complemented by systemic endocrine factors released into circulation by subsequently trained LB [24]. For example, the change in myostatin-follistatin levels is almost twice as large if both UB and LB are exercised together compared with any of these regions alone, shifting the ratio in the advantage of follistatin [15]. Therefore, a limited body of research suggests the potential of the VST to arise from an endocrine-producing (i.e. high-volume) LB workout to strength-trained (i.e. high-resistance) multiple UB muscles in recreational male population under the condition that these (UB) muscles are exercised either before the leg session [21], or simultaneously [54], but not afterwards [44] so to avoid enervation of the neural impulses toward the targeted fibres. Notwithstanding relatively speculative conclusions, these investigations offer valuable insights into the strategies for taking advantage of the VST phenomenon magnifying strength gains via effective training sequencing. More research is needed to evaluate the magnitude of these adaptations with different exercise interventions involving different sexes and athletes from different sports.

### Vertical Strength Transfer with the Exercise of One Body Segment

While uncertainties may exist around the main contributors to the VST when both body segments are concurrently trained, it is highly unlikely to suggest any other but neurological adaptations explaining strength increases in the untrained body parts as a response to the training of remote body parts. The main findings from this section suggest that neurophysiological responses to LB exercise may be important in increasing and preserving strength gains for the muscles in upper extremities [22, 23, 31, 50, 52].

Neural adjustments to strength training generally include two major sites: (1) the CNS with the modified corticospinal excitability and intracortical inhibition [77, 148, 149] and (2) the peripheral nervous system with altered motor unit behaviour reflected through the increased discharge rate, rate coding, synchronisation, recruitment and reduced coactivation of antagonists [150–153]. Any of the aforementioned mechanisms may explain the dissipation of strength from the trained to the untrained muscle groups. However, it is somewhat intuitive to assume that UB resistance training will not have the capacity to alter neural responses in sufficient amounts to modify LB strength without the local exercise stimulus owing to the difference in the size of the muscles between these two regions. For example, the cross-education effect is greater in lower extremities [19, 20], and it is likely that larger muscles require more intense neural signals to result in pronounced neuromuscular adaptations. This was suggested with the findings by Kraemer et al. [45], where LB power and strength were barely impacted by the UB strength training programme alone, whereas UB power expression was augmented by the addition of high-resistance LB exercises. Therefore, a complementary result for the neural drive towards the LB muscles may only be possible when both regions are concurrently trained, and this is yet to be affirmed with future investigations.

To further evaluate the role of the CNS for the VST, this review included studies that examined strength changes in the upper limbs after interventions that exclusively involved LB exercises. They all resulted in significant strength increases for children [50, 51], older populations [31, 52] and adults [22, 23]. These improvements likely occurred under different adaptive mechanisms depending on the age categories. For instance, novel patterns of movements may have provoked enhanced global neural responses for youngsters [154], followed by superior neurophysiological adaptations [155, 156] that had the potential to preserve strength gains even after a 4-week detraining period [51]. In contrast, authors from the studies with older adults [31, 52] suggested biochemical factors known as exerkines inducing a crosstalk between remote tissues and causing strength improvements in the upper extremities. However, despite the elevated levels of superoxide dismutase activity [31], which is a proven therapeutic agent [32], it may be more feasible to suggest that the activation of UB muscles during cycling and strength training (to maintain balance) caused increases in handgrip strength for inactive older adults. Positive results were also found in a study by Aman et al. [23], where middle-aged women’s upper limb strength significantly improved (33.9–58.3% increase) after LB resistance and “neuromuscular exercises” (i.e. balance, agility, strength) across a 12-week period. It is possible that repetitive LB contractions supported the downregulation of inhibitory feedback by the afferent nerves [157], whose purpose is to deactivate alpha motor neurons of the contracted muscle when high forces are applied [158]. With sustained muscular activation, these signals are inhibited [157], and favourable alterations in spinal reflexes have been proven to occur [77]. This would result in an enhanced motor drive by the CNS [159], supporting strength improvement in the untrained muscles. All these mechanisms look important for the further exploration of the VST with its potential to increase strength on the account of neurological enhancements. For example, UB strength training may have a complementary neuromuscular effect with the facilitation of leg-dominant high-intensity actions such as sprinting [160] or jumping [34]. Nonetheless, further research involving athletic populations is necessary before making conclusive interpretations based solely on the findings from untrained individuals.

“Cross-education’’ is a well-established phenomenon that explains strength gains in the untrained limb after the exercise of the contralateral limb due to the modified neural plasticity [105, 148]. Two models proposed to interpret cross-education are the “bilateral access model,’’ which describes that unilaterally created motor engrams can be utilised bilaterally, and the “cross-activation model’’, which explains that unilateral contractions are driven by both the ipsilateral and contralateral motor cortices [148]. While strength training is proposed to be governed by cross-activation, more complex tasks have been suggested to promote bilateral access [105]. Magdi et al. [22] tried to maximise neuromuscular responses by taking advantage of both models, organising participants to attend unilateral leg training sessions where they were required to perform strength-based and proprioceptive-based leg exercises. The idea was to stimulate the VST from the trained leg to the untrained arm. Authors intended to magnify this effect by emphasising eccentric leg contractions [161–164] and got a remarkable transfer from the exercised lower limb to the non-exercised upper limb (MVIC increase: men 14.7%, women 69.4%; biceps 1-RM increase: men 10.5%, women 20.6%; power increase with low loads: men 59.0%, women 72.6%, medium loads: men 47.1%, women 60.8%, and high loads: men 19.6%; women: 53.3%) [22]. Unfortunately, no tests were conducted on the contralateral arm to examine whether the VST dissipated to this arm as well. The findings from this study [22] highlight the potential of unilateral eccentric-based resistance training to increase force and power production in the ipsilateral remote limb. Further research should explore the extent to which this type of exercise may be utilised to stimulate neurological responses with the application to the recovery from injury. It could be particularly beneficial for athletes who need to preserve strength in injured extremities during the rehabilitation process in order to be ready to perform again in shorter time periods.

The main limitation of this review was the lack of studies investigating the effect of UB resistance training on LB strength or power-generating adaptations, which required more speculative discussion using the reverse order of transfer (i.e. LB to UB). Furthermore, VST was mostly discussed in relation to the recreational population, which is arguably more prone to neurological alterations compared with professional athletes.

## Conclusions

The purpose of this systematic scoping review was to investigate the existence of the VST phenomenon as a response to different exercise configurations and identify potential mechanisms underpinning its occurrence. The findings from the review highlight some important points: (1) the addition of UB strength training to LB endurance training may help preserve leg muscle morphology and power generation in LB. This exercise combination may also contribute to strength gains in LB muscles for older population; (2) high-volume or BFR type of LB resistance exercise may facilitate strength training adaptations for: (a) previously or subsequently trained single muscle group from UB (e.g. elbow flexors) and (b) previously or simultaneously trained multiple muscle groups from UB (i.e. upper torso with upper limbs); and (3) strength exercise sessions for LB muscles could improve strength in untrained upper extremities on the basis of an increased neural drive. More research is needed to elucidate whether the VST phenomenon could help to enhance performance for an athletic population and potentially preserve high-intensity force production in injured extremities as a result of exercise with healthy extremities. This phenomenon might also be important for the preservation of power-generating capacity in endurance-trained LB muscles by strength-trained UB muscles, thereby also benefitting specific athletic populations (e.g. soccer players), for which future investigations are warranted.
